# Treatment of La Grippe

**Published:** 1892-04

**Authors:** 


### HALL'S
### JOURNAL OF HEALTH

TRUTH DEMANDS NO SACRIFICE; ERROR CAN MAKE NONE.

Vol. 39. APRIL, 1892. No. 4.

### TREATMENT OF LA GRIPPE.

In our last issue we published a very comprehensive article on La Grippe.

The following equally comprehensive article on its treatment by an eminent English physician will be found a great help to the afflicted of this dangerous and frequently fatal malady.

Dr. Hunter says :—The three cardinal points of management are all that need be borne in mind to rob this disease of its worst terrors, and secure to each sufferer a speedy deliverance from the fever, and an early restoration to health. I claim no originality for my method. Though in several respects singular—that is to say, contrary to the general practice—it is only what might naturally be followed by any intelligent person who was moderately instructed on matters of health and in the principles of hygiene, and free from bias of any kind. An essential, if simple, part of the treatment lies not so much with the doctor but pertains rather to the patient. Nature demands perfect rest in this disease, and points her demand with imperious finger; yet it seems that more than ordinary sense is needed to secure prompt and thorough compliance. There is a strong predilection in the human mind for "doing something." And what with doing the usual work until complete prostration renders work an impossibility, and then seeking to undo that prostration by stimulating foods and drinks, all that is serious in the situation arises, and the patient at length finds himself utterly undone.

To concede everything, and at once, to nature's demand for rest from the very first, is the only way of securing victory.

The first rule, therefore, is to take to bed immediately on the earliest indication of a seizure, viz., symptoms of a severe chill pervading the whole body. Once in bed, let the patient keep there until the last symptom has gone. This rule is absolute, and any tardiness in compliance therewith will assuredly vitiate every other step in the series of observances.

The second rule is to drink copiously of water, plain or aerated, flavored or otherwise, as fancy may suggest, but free, or all but free, from sugar, milk or alcohol. A quart of liquid a day, at least, should be taken, and as much more as can be managed. The blood wants washing out, as will be made plain later on. Water gruel, barley-water, toast-water, celery-water, apple or orange-water, or lemon juice and water, all unsweetened, or sweetened with saccharine or glycerine, in place of sugar—these are of the first importance, present and future.

The third rule is that no strong "support" be taken during the acute stage, the first three or four days—that is to say when the temperature is up. Beef-tea, chicken soup, meat jellies, and "stimulants" of all kinds must be absolutely abjured. They will but stimulate the disease, re-enforce the enemy, and work havoc there and thereafter. Of this I have become fully assured, and I would endeavor to impress this assurance to the utmost, because it will have to encounter considerable assurance to the contrary, as I am well aware.

Moreover, there must be no sedatives, no antipyrin for the pains and the prostration, and the worse than pain, those sleepless nights and restless days at the outset. These symptoms are otherwise provided against—that is to say, as far as is compatible with the ultimate welfare of the patient.

The feeding needs special attention. For the brief term of the febrile stage, a breakfastcupful of oat meal, or one or other of the many infants' foods, preferably Bovinine, administered every two or three hours all the clock round, and with what gentle pressure may be requisite, will amply suffice. Such foods should be selected as are not unpalatable, though made with water instead of milk, and seasoned with salt in place of sugar. A little milk may be added at the time of eating, and some pieces of dry toast, or a rusk or two, or plain biscuit. The fever may meanwhile pass all the quicker for a brief vacation to stomach and liver.

A couple of days of comparative fasting are not amiss for the average subject, one who has been well fed prior to the seizure ; and too much pressure to take food may be quite malapropos until after defervescence has become complete, that is, when the fever has completely gone, and the temperature becomes natural. Thereafter the rule is, of course reversed, and to feed up is the one great duty during convalescence. Grapes, baked apples, and an occasional orange, are to be recommended all through the attack. Lemons are specially desirable, the juice being positively remedial in this conjuncture, and much more than a mere refreshment. These should be taken freely all through the acute stage, two, three or four lemons a day, or their equivalent in lemon or lime juice from the bottle. A high colored, scanty urine is the one unerring indication for them; and until that changes to a secretion both clear and copious, too much lemon or lime juice cannot be administered,—of course, always diluted, the more freely the better.

And this brings me to emphasize, for my professional brethren, the one great guiding indication, pathological and therapeutic, of this variety of influenza, the *lithiasis* above referred to, that red, yellow, or brown deposit in the urine, notable even to the uninstructed. I is a constant feature of this disease. An early appreciation of the importance of this fact as a guide in its direction, has stood me in very good stead. While the urine, on cooling, affords the deposit above mentioned, the system is overcharged as regards the blood with effete and injurious matters, and in this state can derive no real advantage from stimulants and strong animal broths. On the contrary, the patient must suffer from their ill-timed addition to a blood already all too morbid with nitrogenous matters; while to a liver, congested as that organ always is with the blood in this state, alcohol is an irritant of the worst kind, and will assuredly perpetuate and increase the very trouble it is given to relieve.

